# Functional Implications of Multiple IM30 Oligomeric States

**DOI:** 10.3389/fpls.2019.01500

**Published:** 2019-11-21

**Authors:** Carmen Siebenaller, Benedikt Junglas, Dirk Schneider

**Affiliations:** Department of Pharmacy and Biochemistry, Johannes Gutenberg University Mainz, Mainz, Germany

**Keywords:** IM30, Vipp1, PspA, thylakoid membrane, membrane fusion, membrane stabilization, membrane dynamics, heat shock proteins

## Abstract

The inner membrane-associated protein of 30 kDa (IM30), also known as the vesicle-inducing protein in plastids 1 (Vipp1), is essential for photo-autotrophic growth of cyanobacteria, algae and higher plants. While its exact function still remains largely elusive, it is commonly accepted that IM30 is crucially involved in thylakoid membrane biogenesis, stabilization and/or maintenance. A characteristic feature of IM30 is its intrinsic propensity to form large homo-oligomeric protein complexes. 15 years ago, it has been reported that these supercomplexes have a ring-shaped structure. However, the *in vivo* significance of these ring structures is not finally resolved yet and the formation of more complex assemblies has been reported. We here present and discuss research on IM30 conducted within the past 25 years with a special emphasis on the question of why we potentially need IM30 supercomplexes *in vivo*.

## Im30 Is Involved in Tm Protection and Remodeling

The thylakoid membranes (TMs) of chloroplasts and cyanobacteria harbor the complexes of the photosynthetic electron transfer chain. The emergence of TMs in cyanobacteria is evolutionary coupled to the development of the inner membrane-associated protein of 30 kDa (IM30)/vesicle-inducing protein in plastids 1 (Vipp1)-protein (Vothknecht et al., 2012), and while Vipp1/IM30 is clearly linked to the biogenesis/maintenance of TMs, its exact physiological function still is unclear. As this protein appears to be essential for proper development of a functional TM system and therefore the whole photosynthetic apparatus, clarifying the involvement of Vipp1/IM30 in TM biogenesis/maintenance is vital to understand and eventually reconstruct the photosynthetic machinery, which is the major energy source for life on earth.

The inner membrane-associated protein of 30 kDa (IM30) was first described in 1994 as a protein with a dual localization at the inner envelope (IE) and at TMs in *Pisum sativum* chloroplasts (Li et al., 1994). In 2001, homologs of this protein have been identified and characterized in *Arabidopsis thaliana* and the cyanobacterium *Synechocystis* sp. PCC 6803 (from now on: *Synechocystis*) (Kroll et al., 2001; Westphal et al., 2001). Due to an apparent deficiency in vesicle formation at low temperatures of *Arabidopsis* Vipp1 depletion mutants, IM30 was renamed to *vesicle inducing proteins in plastids* 1 (Vipp1) (Kroll et al., 2001). In recent years, IM30/Vipp1 has been found to be essential for TM formation and IM30/Vipp1 was suggested to be involved in many processes linked to TM maintenance and/or biogenesis (summarized in Heidrich et al., 2017). As the proposed involvement in vesicle formation was not supported by any additional data, we here name the protein as originally proposed, *i.e.* IM30.

**Figure 1:**
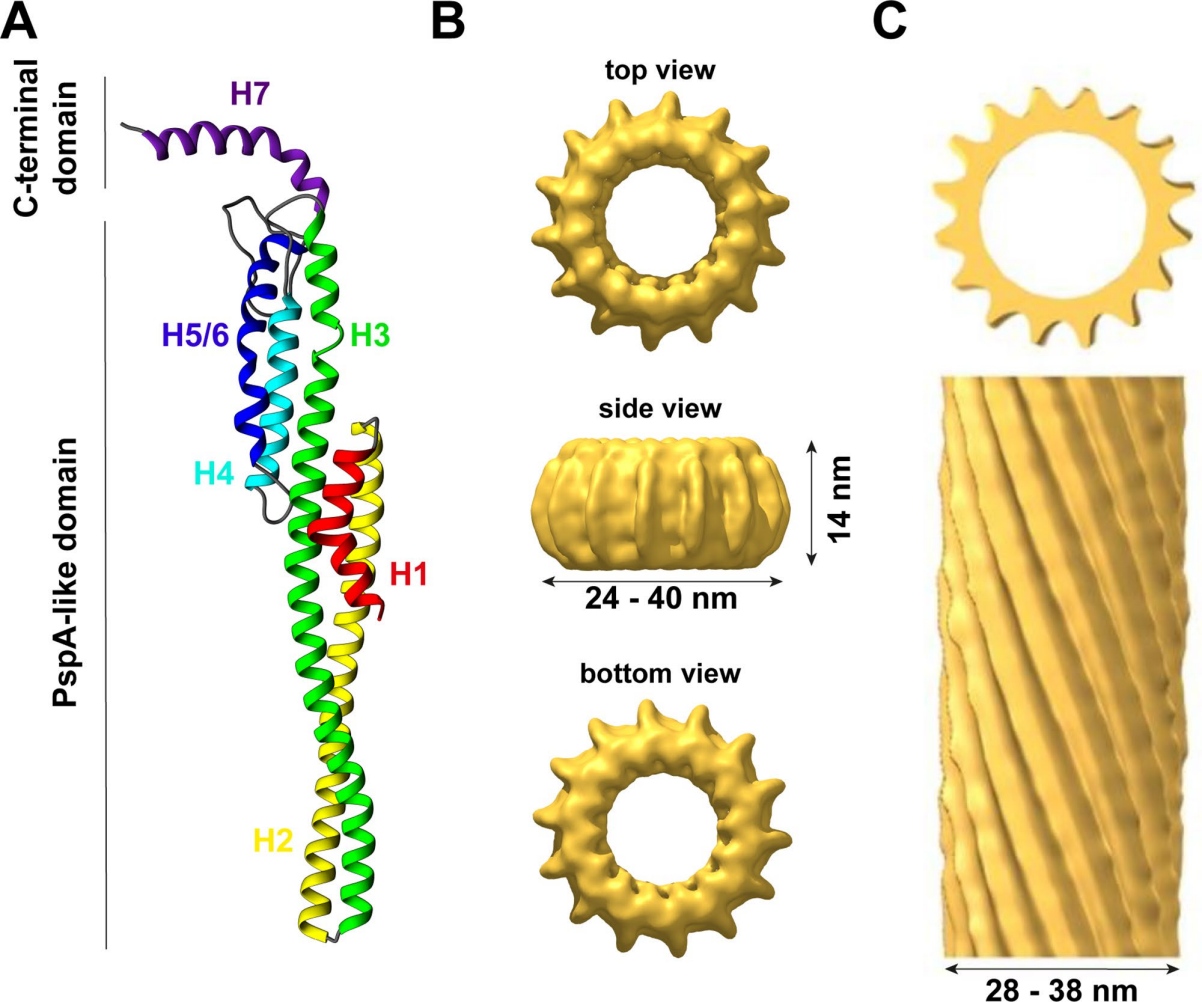
The structure of IM30. **(A)** IM30 is a predominantly α-helical protein with seven helices. Helix 1–6 form the PspA-like domain. IM30 can be discriminated from PspA by a C-terminal extension, which includes an additional helix. **(B)** IM30 forms ring-like homo-oligomers with diameters ranging from 24 to 40 nm and a height of 14 nm (EMDB:3740). **(C)** IM30 forms rod-like structures with typical diameters of 28–38 nm [adapted from (Theis et al., 2019); open-access license http://creativecommons.org/licenses/by/4.0/].

IM30 proteins are conserved amongst almost all photosynthetic organisms (Westphal et al., 2001; Vothknecht et al., 2012), and phylogenetic analyses have revealed that IM30 proteins potentially evolved *via* gene duplication from the bacterial phage shock protein A (PspA) during evolution (Westphal et al., 2001). Although sequence identity (∼30%) and similarity (∼50%) are not too high between PspA and IM30 proteins (Bultema et al., 2010), both proteins appear to share a highly conserved (predicted) secondary structure with an N-terminal core structure of about 220 amino acids consisting of six α-helices (the so-called PspA-like domain, **Figure 1A**). A major structural difference between PspA and IM30 is an extra C-terminal α-helix in IM30 proteins that is connected to the PspA domain *via* an extended linker region (Westphal et al., 2001; Otters et al., 2013). This extra domain of 20–30 aa possibly discriminated PspA from IM30 proteins and potentially causes the specialized functions of IM30 during TM biogenesis/maintenance, which cannot be accomplished by PspA (Westphal et al., 2001; Aseeva et al., 2004; Bultema et al., 2010; Vothknecht et al., 2012). In contrast, IM30 can functionally replace PspA in *E. coli pspA* null mutants (DeLisa et al., 2004; Zhang et al., 2012), suggesting a conserved function of the PspA domain and a more specific function of the C-terminal IM30 domain in TM biogenesis/maintenance gained during evolution. Nevertheless, due to their similarities, PspA and IM30 together form the PspA/IM30 protein family, together with LiaH, a phage shock protein homolog (Wolf et al., 2010).

An outstanding feature of all members of the PspA/IM30 protein family is their ability to organize into large ring-shaped homo-oligomeric (super)complexes (as further discussed below), which have first been described 15 years ago for both, PspA and IM30 (Aseeva et al., 2004; Hankamer et al., 2004) and were later on also identified for LiaH (Wolf et al., 2010). Interestingly, the occurrence of these IM30 supercomplexes appears to depend on the presence of chaperones/chaperonins, which are likely involved in assembly and disassembly of the IM30 supercomplexes (at least *ex vivo*) (Liu et al., 2005; Liu et al., 2007; Gao et al., 2015).

The exact physiological function of IM30 monomers and/or oligomers is still not finally resolved yet. In recent years, potential *in vivo* functions of IM30 have mainly been studied using IM30 depleted or deleted cyanobacteria, algae, or plants. In fact, most studies were performed in the cyanobacterium *Synechocystis* sp. PCC 6803, the green alga *Chlamydomonas reinhardtii* or the higher plant *Arabidopsis thaliana* (**Table 1**). Yet, results obtained after protein depletion were not entirely conclusive. While depleting IM30 in the cyanobacteria *Synechococcus* sp. PCC 7002 or *Synechocystis* sp. PCC 6803 (Westphal et al., 2001; Fuhrmann et al., 2009b; Zhang et al., 2014) as well as in *Arabidopsis* chloroplasts (Aseeva et al., 2007; Vothknecht et al., 2012) resulted in reduced TM networks and a disturbed TM morphology, depleting the protein in *Chlamydomonas* did not affect the TM structure (Nordhues et al., 2012). However, *Chlamydomonas* contains two IM30 paralogs (named Vipp1 and Vipp2), and in Nordhues *et al*. solely expression of one paralog was reduced. Yet, depletion of this paralog resulted already in an altered photosynthetic activity in *Chlamydomonas*, as has also been observed in cyanobacteria, but not in *Arabidopsis*, with photosystem II being affected in *Chlamydomonas* and photosystem I in cyanobacteria (Aseeva et al., 2007; Fuhrmann et al., 2009a; Nordhues et al., 2012; Vothknecht et al., 2012). Noteworthy, in contrast to most other studies, Gao *et al.* describe that depletion of IM30 in *Synechocystis* did lead to a generally reduced photosynthetic activity but not to TM reduction (Gao and Xu, 2009). While these results could indicate different roles of IM30 in different species, even results obtained in the same strain are not conclusive (Gao and Xu, 2009; Fuhrmann et al., 2009b). We believe that the major activity of IM30 remained conserved throughout evolution and that differences were observed due to other species-specific features, e.g., in some photosynthetic organisms certain lipids are essential whereas these can be replaced by other lipids in other species. This has e.g. been well studied in case of sulfoquinovosyldiacylglycerol (SQDG), which can be replaced by phosphatidylglycerol (PG) to some extent in *Synechococcus*, but not in *Synechocystis* or *Arabidopsis* (Aoki et al., 2004; Kobayashi et al., 2015). As IM30 likely interacts with defined lipids and as lipids are crucial building blocks of TMs as well as part of photosystems, the observed differences could be explained by this. Yet, also other species-specific factors are described to be exclusively involved in TM and/or photosystem biogenesis in chloroplasts or cyanobacteria (Anbudurai et al., 1994; Guskov et al., 2009; Umena et al., 2011; Heinz et al., 2016). Yet, within the last 25 years, besides many others (Kroll et al., 2001; Benning et al., 2006; Göhre et al., 2006; Fuhrmann et al., 2009b; Nordhues et al., 2012; Rütgers and Schroda, 2013; Zhang et al., 2014; Walter et al., 2015) (reviewed in more detail in (Heidrich et al., 2017)), two major physiological functions of IM30 have been suggested, which we briefly introduce here:

(i) Membrane protection:

PspA, the major effector of the bacterial phage shock system, is known to have a membrane stabilizing/protecting function, and binding of PspA to membrane surfaces helps to maintain the proton motive force (PMF) (Kleerebezem et al., 1996; Kobayashi et al., 2007; Joly et al., 2010). Due to the high similarity of PspA and IM30, it appears reasonable to speculate that IM30 also has a membrane-stabilizing/-protecting function (Vothknecht et al., 2012; Zhang et al., 2012; Zhang and Sakamoto, 2013; Zhang and Sakamoto, 2015). Indeed, both, PspA and IM30, bind to negatively charged lipid membranes in a curvature dependent manner *in vitro* (Kobayashi et al., 2007; Hennig et al., 2015; McDonald et al., 2015; Heidrich et al., 2016) and IM30 potentially increases the lipid order upon membrane binding (Hennig et al., 2015; Heidrich et al., 2016). While this suggests that the protein preferentially binds to ordered membrane regions (*i.e.* gel-phase membranes), further experimental proof is missing. Besides these *in vitro* observations, it has been observed that heterologous expression of IM30 from *Synechocystis* and *Arabidopsis* can complement deficiencies in a bacterial Δ*pspA* mutant (DeLisa et al., 2004; Zhang et al., 2012) and that IM30 overexpression can increase the heat stress tolerance in *Arabidopsis* (Zhang et al., 2016). As small IM30 oligomers bind with higher affinity to negatively charged membranes than the large oligomeric ring structures (Heidrich et al., 2016), it has been hypothesized that IM30 and PspA rings disassemble on membranes and function as membrane chaperones by forming a membrane protective structure upon membrane binding (Thurotte et al., 2017; Junglas and Schneider, 2018).

(ii) Membrane remodeling:

While IM30 appears to share its membrane-stabilizing/-protecting activity with PspA, IM30 clearly must have acquired additional functions in cyanobacteria and chloroplasts, as PspA is not able to replace IM30 (Westphal et al., 2001; Aseeva et al., 2004; Bultema et al., 2010; Vothknecht et al., 2012). Expression of IM30 appears to be of special importance when cyanobacterial cells are shifted from dark to light growth conditions (Gutu et al., 2018), where photosynthetic organisms need to adapt their photosynthetic apparatus to account for the changing light intensities by dynamic rearrangement of the TM system (Chuartzman et al., 2008; Nagy et al., 2011; Liberton et al., 2013). Such TM rearrangements require extensive membrane remodeling, and a likely candidate catalyzing TM remodeling is IM30. IM30 can induce fusion of liposomal membranes, at least *in vitro* (Hennig et al., 2015; Thurotte and Schneider, 2019), a process that appears to be controlled by the cytosolic Mg^2+^ concentration, as Mg^2+^ directly binds to IM30 and thereby triggers the fusion process (Hennig et al., 2015; Heidrich et al., 2018; Thurotte and Schneider, 2019). This is of special importance in TM-containing organisms, as the cytosolic Mg^2+^ concentration varies in the dark *vs.* light and depends on the photosynthetic activity (Pohland and Schneider, 2019). Thus, the IM30-specific membrane remodeling activity appears to be (indirectly) controlled by light. Besides light, GTP binding and hydrolysis were recently suggested to control the IM30 membrane remodeling function (Ohnishi et al., 2018), although IM30 does not contain a canonical G-domain and GTP is not required *per se* for membrane binding and liposome fusion (Hennig et al., 2015). Noteworthy, the suggested membrane-stabilizing and the membrane-remodeling activity of IM30 on the first view contradict each other, at least in part, as membrane fusion processes typically involve membrane destabilization. However, both functions might be relevant *in vivo*, as Mg^2+^-release or binding to IM30 could control the respective activities (Junglas and Schneider, 2018).

**Table 1:** The IM30 supercomplex structures in different species.

Organism	Ultrastructure *in vitro*	Diameter	Ultrastructure *in vivo*	Size
*Synechocystis* sp. PCC 6803	Mostly rings, (rods) ^[1,2]^	25–33 nm ^[2]^	At membranes: dynamic and static punctae ^[8,9]^	100–300 GFP molecules estimated: 100 ± 25 nm ^[8]^
			In the cytoplasm: diffuse particles ^[8,9]^	
*Arabidopsis thaliana*	Mostly rings, (rods) ^[3,4]^	40 nm ^[3]^	At membranes: static clusters ^[4,10]^ In the stroma: mobile IM30 particles ^[4,10]^	<0.2–1.5 µm ^[4]^
*Chlamydomonas reinhardtii*	Mostly rods, rings ^[5,6]^	28–37 nm ^[5]^	*n.a.*	*n.a.*
*Triticum urartu*	Mostly rings, (rods) ^[7]^	∼30 nm ^[7]^	*n.a.*	*n.a.*

[1] (Fuhrmann et al., 2009a), [2] (Saur et al., 2017), [3] (Aseeva et al., 2004), [4] (Zhang et al., 2016), [5] (Liu et al., 2007), [6] (Theis et al., 2019), [7] (Gao et al., 2017) [8] (Bryan et al., 2014), [9] (Gutu et al., 2018), [10] (Zhang et al., 2012). n.a. = data not available.

## Im30 Structure: What Do We (Not) Know So Far?

### The Monomer Structure

Thus far, the structure of the IM30 monomer is still elusive. The monomer is supposed to have a highly α-helical structure (∼80% α-helix) with six helices separated by short linker regions (Fuhrmann et al., 2009a; Gao et al., 2015). Additionally, helix 7 is separated from the PspA(-like) domain by an extended flexible linker (Otters et al., 2013). All these assumptions are based on secondary structure predictions but are supported by CD-spectroscopy and FTIR measurements (Fuhrmann et al., 2009a; Gao et al., 2015; Heidrich et al., 2018). Recently, a model of the IM30 monomer has been reported (see **Figure 1A**) that is based on the X-ray structure of a PspA fragment (amino acids 1–144) (Osadnik et al., 2015) and homology modeling (Saur et al., 2017). The X-ray structure of the PspA fragment revealed that helix 2 and 3 form an extended hairpin coiled-coil structure (Osadnik et al., 2015), which appears to form the structural core of the PspA domain. Suggested structural and functional roles of each helix were discussed in more detail recently (Heidrich et al., 2017). Studying truncated IM30 variants allowed to deduce the involvement of individual helices in protein oligomerization (Otters et al., 2013; Gao et al., 2015; Thurotte and Schneider, 2019). Based on these analyses, helix 2 and 3 form the structural core of IM30 that is crucial for supercomplex formation, but by itself does exclusively form monomers (Thurotte and Schneider, 2019). Adding helix 1 and 4 to the structural core enables the formation of dimers (Thurotte and Schneider, 2019) or intermediate-sized oligomers (800 kDa) (Gao et al., 2015), but not of ring-shaped supercomplexes. At minimum, helices 2–6 are required for the formation of stable ring/supercomplex structures (Thurotte and Schneider, 2019). Hence, the helix 2/3 coiled-coil apparently interacts with helix 4 and/or 5/6 in the supercomplexes.

### Small Oligomers

In solution, isolated IM30 has a strong tendency to spontaneously form homo-oligomeric supercomplexes, as further discussed below. Yet, a minor fraction of the protein still forms small oligomers (mostly tetramers and dimers), and also the basic building block of the ring complex appears to be an IM30 tetramer (Liu et al., 2007; Fuhrmann et al., 2009a; Saur et al., 2017). Although some low-resolution data of the structure of the supercomplexes are available, essentially nothing is known about the structure of the small oligomers. Thus far, solely a hypothetical model describing the organization of the monomers in the ring structure, including the tetrameric building block, was suggested (Saur et al., 2017) (**Figure 1A**).

### IM30 Supercomplexes

In 2004, PspA (Hankamer et al., 2004) and IM30 (Aseeva et al., 2004) were reported to form homo-oligomeric supercomplexes with ring-like structures and molecular masses exeeding 1 MDa. In the following 15 years, one main aspect of the research on IM30 was to analyze the structure and implications of these large supercomplexes.

In various experiments, involving size exclusion chromatography (SEC), BN-PAGE, and sucrose gradient centrifugation, members of the PspA/IM30 family were found to mainly organize into high molecular mass complexes in solution, besides a small fraction of dimers/tetramers (Aseeva et al., 2004; Liu et al., 2005; Liu et al., 2007; Fuhrmann et al., 2009a; Gao et al., 2015; Heidrich et al., 2016; Saur et al., 2017). This has been observed for IM30 in cellular extracts of cyanobacteria and chloroplasts of green algae or vascular plants, but also for heterologously expressed and purified proteins (Aseeva et al., 2004; Liu et al., 2005; Liu et al., 2007; Fuhrmann et al., 2009a; Gao et al., 2015; Heidrich et al., 2016; Gao et al., 2017; Saur et al., 2017). As no other proteins appear to be necessary for IM30 oligomerization, the complexes identified in cellular extracts likely represent homo-oligomeric assemblies.

The size of the *EcoPspA* complex was determined via SEC to be ∼1 MDa, indicating that the complex contains 36–37 subunits (Hankamer et al., 2004). For isolated IM30, the molecular mass was determined to be >1 MDa for *Arabidopsis* IM30 (*Ara*IM30) (Aseeva et al., 2004; Otters et al., 2013), for *Chlamydomonas* IM30 (*Cr*IM30) (Liu et al., 2007; Gao et al., 2015) as well as for two IM30 paralogs encoded in Tricum urartu (Gao et al., 2017). The size of *Synechocystis* IM30 (*Syn*IM30) was estimated via SEC to be about 1600–2000 kDa (or even higher) (Fuhrmann et al., 2009a). For the homologous LiaH protein of *B. subtillis*, a molecular mass of at least 1.25 MDa was determined via SEC (Wolf et al., 2010). As these high molecular mass supercomplexes elute in the void volume or close to the void volume in most SEC experiments and as a compact globular shape is assumed in SEC analyzes, which deviates from the partially hollow ring structure of IM30/PspA rings, the determined masses have to be taken with caution. In fact, from a recent 3D-reconstruction of IM30-rings, a molecular mass of about 1.5–2.5 MDa could be roughly estimated by using the volume/shape of the complex (Saur et al., 2017). However, the low resolution of this 3D-reconstruction makes it difficult to set the correct contour level for an exact determination of the volume and thus the exact mass.

Ring-shaped supercomplexes have been observed multiple times via negative stain electron microscopy for purified PspA (Hankamer et al., 2004), LiaH (Wolf et al., 2010) and IM30 from different organisms, involving *Arabidopsis*, *Chlamydomonas* and *Synechocystis* (Aseeva et al., 2004; Liu et al., 2007; Fuhrmann et al., 2009a; Gao et al., 2015). Thus far, the prevailing thesis is that PspA rings solely occur with 9-fold rotational symmetry (from *E. coli*), indicating a 4x9 (= 36 monomers) structure, which is in agreement with the molecular mass estimated via SEC. These *Eco*PspA rings have a diameter of about 20 nm and a height of 8–11 nm (Hankamer et al., 2004). However, *Eco*PspA rings with different diameters have also been observed, although they have not been further characterized (Male et al., 2014). The symmetry and number of monomers of the LiaH rings were identical with the PspA rings described by Hankamer et al. (2004); yet a ring diameter of 25 nm has been determined (Wolf et al., 2010).

In contrast to the supposedly homogeneous PspA and LiaH supercomplex structures, the IM30 ring dimensions are clearly highly variable. The first electron micrographs of heterologously expressed *Ara*IM30 revealed ring-shaped particles with a diameter of about 40 nm and a height of about 14 nm (**Figure 1B**) (Aseeva et al., 2004). Subsequent more detailed analysis of *Cr*IM30 and *Syn*IM30 revealed a heterogeneous size distribution with rings having diameters of at least 28–37 nm (*Cr*IM30) (Liu et al., 2007) and 25–33 nm (*Syn*IM30), respectively, resulting in a calculated number of monomers per ring ranging from 48–72 (Fuhrmann et al., 2009a; Saur et al., 2017). While the ring diameter clearly is variable, a constant height of 13–15 nm was observed for all *Syn*IM30 ring structures (Saur et al., 2017). Most electron micrographs of IM30 exhibit a pronounced spike architecture, giving rise to a very well defined rotational symmetry (at least 7 up to 22fold) (Saur et al., 2017). Interestingly, the 3D-reconstructions of the *Syn*IM30 rings suggest that the rings are polar and have two distinct sides (ring top and bottom side) (**Figure 1B**), with the monomers likely being ordered unidirectional in the ring structure (Saur et al., 2017), possibly enabling the rings to interact with two different interaction partners. This perfectly supports the idea of IM30 rings beeing able to bind/fuse two different membrane surfaces, e.g. different TM sheets or the cyanobacterial cytoplasmic membrane (CM) with the TM (Saur et al., 2017).

### Rod-Like Structures

Besides isolated rings, in electron micrographs of purified IM30 and PspA also double rings and elongated rod-like structures were identified (**Figure 1C**), the latter appear to form via stacking of multiple IM30/PspA rings (Liu et al., 2007; Fuhrmann et al., 2009a; Male et al., 2014; Gao et al., 2015; Saur et al., 2017; Thurotte and Schneider, 2019).

While the formation of rod-like structures is a common feature of IM30/PspA family members, the preference for rings vs. rod-like structures appears to depend on the species (see **Table 1**). *Syn*IM30, the most intensely studied member of the IM30/PspA family in terms of protein structure, does only occasionally form rod-like structures (Fuhrmann et al., 2009a; Saur et al., 2017). Yet, formation of double ring structures is induced by Mg2+-binding to IM30, which alters the surface properties of individual IM30 rings (Heidrich et al., 2018). Additionally, increased formation of rod-like structures has been observed upon removal of the C-terminal helix 7 from *Syn*IM30 (Hennig et al., 2017). This observation suggests that PspA (and/or the PspA core of IM30 proteins) might be more prone to the formation of rod-like structures as they do not contain the (IM30-specific) C-terminal extension. PspA rings were initially observed and analyzed in the presence of chaotropic salts (Hankamer et al., 2004), which might hinder rod formation or disassemble PspA rods. In fact, extensive formation of rod-like structures has recently been reported for *Eco*PspA (Male et al., 2014). Furthermore, truncation of the *Syn*IM30 helix 1 also resulted in an increased formation of rod-like structures (Thurotte and Schneider, 2019), suggesting that helix 1 and helix 7 negatively control rod-formation in *Syn*IM30. Indeed, the removal of helix 1 and helix 7 in combination resulted in the exclusive formation of rod-like structures in the case of *Syn*IM30 (Thurotte and Schneider, 2019). This might be due to the removal of steric barriers inhibiting rod-formation of the wt protein. Interestingly, helix 7 is known to protrude out of the ring core structure (Otters et al., 2013; Heidrich et al., 2018) and thereby may hinder ring-ring contacts at one side of the ring. However, both helix 1 and 7 seem to have an intrinsic propensity to be unstructured (Osadnik et al., 2015; Zhang et al., 2016; Hennig et al., 2017; McDonald et al., 2017). Thus, they occupy a large conformational space, which might explain why they can create a steric hindrance for ring/supercomplex formation.

Notably, while *Ara*IM30, as well as the two IM30 paralogs of *Triticum urartu*, also appear to have a rather weak tendency to form rod-like structures (to the best of our knowledge, as the experimental evidence on these structures is limited (Otters et al., 2013; Zhang et al., 2016; Gao et al., 2017)), *Cr*IM30 has a pronounced tendency to form rods (Liu et al., 2005; Liu et al., 2007; Theis et al., 2019) (**Table 1**).

Also, deletion of helix 1 has different effects on the ultrastructure of IM30 from different species: While deletion of helix 1 in *Syn*IM30 clearly induced rod formation, deleting helix 1 from *Eco*PspA (Jovanovic et al., 2014) as well as from *Ara*IM30 (Otters et al., 2013; Ohnishi et al., 2018) did even abolish formation of large oligomers (including rings and rods), and thus here helix 1 appears to be essential for the formation of large oligomers as well as rings or rods. In contrast, removal of helix 1 from *Cr*IM30 did not abolish formation of large oligomers, albeit the oligomers appear not to have the prototypical ring structures anymore (Gao et al., 2015). Thus, the exact role of helix 1 for supercomplex formation appears to be species-dependent and has to be analyzed in more detail. Nevertheless, helix 1 and 7 are crucially involved in (de)stabilization of IM30 supercomplexes.

However, while also the physiological relevance of the rod-like structures is not at all clear yet, it has been hypothesized that they might be part of cytoskeleton-like elements with microtubule-like structures (Liu et al., 2007; Rütgers and Schroda, 2013). Recently, it has been shown that *Cr*IM30 rods can engulf phosphatidylinositol phosphate-containing membranes (Theis et al., 2019), and thus, the rod-like structures could well be part of the membrane remodeling machinery of IM30.

## The Structure of Im30 Changes Dynamically *in Vivo*

The *in vivo* structure of IM30 is still enigmatic. It has been shown via fluorescence microscopy that GFP-tagged IM30 forms large clusters close to the TMs in chloroplasts and cyanobacterial cells, seen as punctae (Bryan et al., 2014). These punctae are called “functional Vipp1 particles” (FVPs) in chloroplasts (Zhang et al., 2016) (see **Table 1**). Additionally, GFP-tagged IM30 has also been identified at the CM and in the cytoplasm of *Synechocystis* (Bryan et al., 2014). Importantly, the localization of IM30 in *Synechocystis* changes when cells are transferred from low-light (LL, 8 µE m-2 s-1 intensity) to high-light (HL, (600 µE m-2 s-1 intensity) conditions. At LL, the majority of IM30 clusters were found at the cyanobacterial CM, whereas under HL conditions the total number of IM30 puncta strongly increased and the IM30 puncta preferentially form at the TM (Bryan et al., 2014). This dynamic relocalization of IM30 in *Synechocystis* has been investigated more extensively by Gutu et al. At standard light conditions (100 µE m-2 s-1 intensity), *Syn*IM30 was identified in two fractions: (i) a diffuse uniformly distributed fraction and (ii) short-lived puncta closely associated with highly curved TM regions. Yet, at HL conditions, IM30 puncta stably form at and associate with TMs (Gutu et al., 2018), and potential implications of this relocalization were discussed in more detail recently (Junglas and Schneider, 2018). A similar mobility of FVPs has also been observed in chloroplasts when protoplasts from *Arabidopsis* were treated with hypotonic stress (Zhang et al., 2016). As all members of the IM30/PspA family appear to be localized in discrete punctae associated with (probably stressed) cellular membranes (Engl et al., 2009; Yamaguchi et al., 2013; Domínguez-Escobar et al., 2014) the transient formation of clusters at defined membrane regions might be linked to the primordial PspA-function, *i.e.* membrane protection/maintenance.

However, the question arises, how IM30 is structured in these clusters? Unfortunately, the resolution of conventional fluorescence microscopy is limited to roughly 200 nm. Thus, single IM30 rings with typical diameters of 30 to 40 nm will be hardly detectable. Nevertheless, it has been suggested that the so-called FVPs in chloroplasts represent IM30 rings or clusters of IM30 rings (Zhang et al., 2016). In fact, the observed clusters have estimated maximal diameters of <0.2–1.5 µm (Zhang et al., 2016) and are thus too large for single IM30 rings and may consist of assemblies of multiple IM30 rings (**Table 1**). Notably, the IM30 puncta observed in *Synechocystis* are much smaller than the FVPs (100 ± 25 nm) and contain about 100–300 IM30 molecules (Bryan et al., 2014) (**Table 1**). Thus, they would consist of at least two to five rings, assuming an average monomer content of the rings of about 60. Taking into account the roughly estimated shape of these puncta, it is rather unlikely that they are formed by rod-like structures, but by multiple IM30 rings sitting next to each other (Junglas and Schneider, 2018). However, it is hard to imagine how membrane attached IM30 rings can stabilize lipid bilayers. Yet, as small IM30 oligomers and/or monomers have a higher membrane binding affinity than rings (Heidrich et al., 2016), it is reasonable to assume that IM30 rings disassemble upon membrane binding. Monomers or small oligomers may then form a protein network on membrane surfaces, similar to the clathrin-like structure that has been described for *Eco*PspA (Standar et al., 2008; Thurotte et al., 2017; Junglas and Schneider, 2018). The assumption that IM30 does not remain ring-structured upon membrane binding is further supported by the recent notion that IM30 rings were not found by template matching in tomograms of *Synechocystis* cells at or close to the highly curved TM ends (Rast et al., 2019), *i.e.* at the regions where the clusters have been identified via fluorescence microscopy. Furthermore, while not being genuine proof, up to the present day IM30 rings have, to the best of our knowledge, never been observed in any study of isolated TMs via electron microscopy or atomic force microscopy, despite the large ring dimensions (Olive et al., 1981; Kirchhoff et al., 2004; Kirchhoff et al., 2008b; Engel et al., 2015; Kowalewska et al., 2016; Casella et al., 2017; MacGregor-Chatwin et al., 2019; Wietrzynski et al., 2019). Hence, we conclude that the observed clusters most likely are not formed from IM30 rings sitting on membrane surfaces. However, the diffuse particles observed by Gutu et al. potentially represent single IM30 rings in solution (Gutu et al., 2018). Unfortunately, for the diffuse particles, no dimensions were given, possibly because the diffuse particles were too small and too mobile.

## Dynamic (Dis)Assembly of Im30 Is Mediated by Chaperones

**Figure 2:**
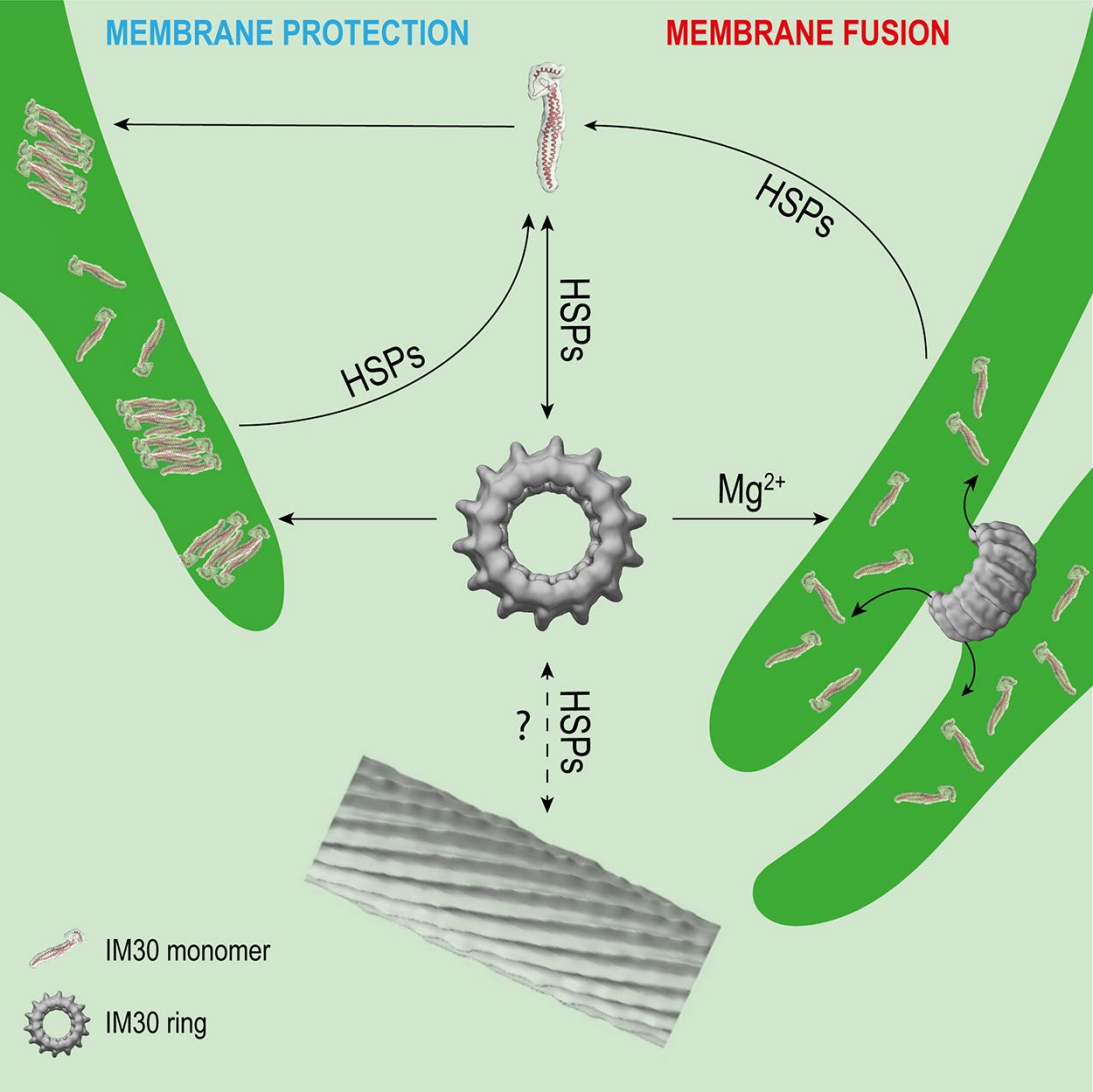
Potential interactions of IM30 with membrane and HSPs. IM30 interacts with the TM as a ring and/or monomer. The monomers possibly rearrange on the TM to form a membrane-protecting structure. In presence of high amounts of Mg^2+^, the IM30 ring is able to fuse adjacent membranes, which might involve dissociation of the ring into membrane-bound small oligomers/monomers. HSPs may detach the monomers from the membrane and trigger homo-oligomerization and ring formation. The physiological relevance of IM30 rod-like structures is unclear so far. However, HSPs have been shown to disassemble IM30 rods in the presence of ATP *in vitro*. (Rod structure adapted from (Theis et al., 2019); open-access license http://creativecommons.org/licenses/by/4.0/).

As observed in the before mentioned *in vivo* studies, the oligomeric state of IM30 appears to be highly dynamic. This dynamic behavior likely involves the activity of chaperones, which have been identified to interact with IM30 proteins (**Figure 2**). In *Chlamydomonas rheinhardtii*, IM30 was shown to associate with HSP70 chaperones and the co-chaperones CDJ2 and CGE1 in ATP-depleted cell extracts (Liu et al., 2005; Liu et al., 2007). These interactions were thereafter confirmed in solubilized membrane fractions (Heide et al., 2009). Additionally, HSP90 was identified as a supplementary interaction partner of the IM30/HSP70 complex in *Chlamydomonas* (Heide et al., 2009) and in *Arabidopsis* chloroplasts (Feng et al., 2014). In *Synechocystis*, the two HSP70 chaperones DnaK2 and DnaK3 (Rupprecht et al., 2007; Rupprecht et al., 2008; Rupprecht et al., 2010), as well as the HSP60 chaperonin GroL1, were shown to interact with IM30 (Bryan et al., 2014).

Interaction of IM30 with different chaperones significantly impacts the oligomeric state of IM30 (**Figure 2**). Although IM30 was found in the IM30/CDJ2 complex in a high- and low-molecular-weight fraction (> > 670 kDa and <230 kDa) in ATP-depleted *Chlamydomonas* cell extracts, it was only part of an intermediate-size molecular-weight fraction (about 670 kDa) in ATP-supplemented cell extracts (Liu et al., 2007), clearly suggesting an ATP-dependent assembly and/or disassembly, as expected when ATP-dependent chaperones are involved. Further analyses showed that also heterologously expressed IM30 can be assembled and disassembled by the HSP70-chaperone machinery in an ATP-dependent manner (Liu et al., 2007). Interestingly, the rod-like structures formed by *Cr*IM30 were also disassembled into IM30 rings and possibly smaller oligomers by the HSP70/CDJ2/CGE1 system when ATP was present (Liu et al., 2007). The bacterial HSP70 *Eco*DnaK was able to replace the *Cr*HSP70 protein in presence of CDJ2 and CGE1 (Liu et al., 2007). Interaction of the *Eco*DnaK protein with the *Cr*IM30 full-length protein was observed directly upon induction of heterologous expression of *Cr*IM30 in *E. coli* cells, suggesting that HSP70s generally recognize and stabilize IM30 monomers and assist in the formation of IM30 oligomers and supercomplexes. Likely, HSP70s shield IM30 domains to prevent unspecific aggregation. In fact, *Eco*DnaK binds with high affinity to truncated versions of the *Cr*IM30 proteins that form smaller oligomers but no ring structure anymore (Gao et al., 2015). In intact *Synechocystis* cells, GFP-labeled DnaK2, DnaK3, and IM30 were observed via fluorescence microscopy to colocalize in specific TM regions under HL-conditions, but not under LL-conditions (Bryan et al., 2014). Thus, under (HL) stress conditions, IM30 is potentially recruited to DnaK2 and/or DnaK3-enriched regions close to the membrane that activate IM30 via catalyzing assembly/disassembly (Bryan et al., 2014). Such relocalization of proteins under stress conditions has also been observed for PspA of *Yersinia enterocolitica* which can be found in the cytoplasm and at the CM under normal conditions, whereas it forms large static complexes at the CM under stress conditions (Yamaguchi et al., 2013).

In an *Arabidopsis* mutant lacking *Ara*HSP90.5, the ratio of monomeric IM30 to higher molecular weight-oligomers of IM30 (>1000 kDa) was shifted to the oligomeric form, indicating that HSP90.5 is also involved in disassembly of IM30 supercomplexes, possibly together with HSP70 (Feng et al., 2014).

Taken together, IM30 clearly has an intrinsic propensity to spontaneously form large oligomeric structures. HSP70 and HSP90 chaperones have been shown to catalyze disassembly, but also the assembly of IM30 oligomers (**Figure 2**), at least *ex vivo* and *in vitro*. The chaperones may be required for removal of membrane-bound small IM30 oligomers (**Figure 2**), as has been described for the uncoating of clathrin complexes by auxilin and HSP70 (Ungewickell et al., 1995). Membrane-associated chaperones are potential candidates for this process. After removal from the membrane, small oligomers may then assemble into oligomers in the cytosol, to finally form the typical ring-shaped IM30 structures and complete the recycling process (**Figure 2**). Yet, it remains to be shown which physiological conditions trigger the interaction of IM30 with the chaperones.

## Why Do We Need a Ring?

In several models, a need for the formation of IM30 rings and/or rod-like structures to fulfill their physiological function(s) is implicated. However, the existence and relevance of IM30 supercomplexes *in vivo* still need to be shown, especially because IM30 rings have never been observed in an *in vivo* context so far. But, are IM30 rings really indispensable for the proposed IM30 functions?

### The IM30 Ring Structure Enables Efficient Membrane Chaperoning

Formation of IM30/PspA rings is probably not necessary for the proteins´ membrane chaperoning function, as small oligomers bind with higher affinity to membrane surfaces (Heidrich et al., 2016). This observation has triggered the suggestion that IM30 rings disassemble into smaller oligomers (or monomers) upon/during membrane binding. It has been hypothesized that PspA/IM30 family proteins act as membrane chaperones by forming a stabilizing network on the membrane surface, as discussed above (Thurotte et al., 2017; Junglas and Schneider, 2018). Possibly, such structures have been imaged via GFP-tagged IM30 in living cells as large IM30 assemblies located at the TM (Zhang et al., 2012; Bryan et al., 2014; Zhang et al., 2016; Gutu et al., 2018; Junglas and Schneider, 2018). Also, the oligomeric PspA structures identified by Standar et al., described as a clathrin-like scaffold (Standar et al., 2008), potentially represent *E. coli* membrane patches coated with PspA.

So, what might the ring structure then be good for, especially in the case of PspA and LiaH proteins that are the main effector proteins of the *psp*/*lia* membrane-stress response system, where their major task is to maintain membranes? Homo-oligomeric protein complexes can provide a highly ordered structure and high stability due to the compactness and cooperativity of highly packed monomers. This might protect the monomeric protein against proteolysis and degradation under harsh conditions, which is especially beneficial for stress-response proteins. Indeed, IM30 rings (more precisely the ring core) are relatively protease-resistant and resistant against chemical and thermal denaturation compared to small oligomers (Gao et al., 2015; Heidrich et al., 2018; Thurotte and Schneider, 2019). Furthermore, the surface of a higher-ordered oligomer is relatively small compared to the monomer. This could be a mechanism to control the IM30 activity, as amphiphilic helices, which are prone to interact with membrane surfaces, are exposed solely when the complexes dissociate. Consequently, the IM30 ring could be a storage form of smaller, active IM30 oligomers/monomers to prevent a continuous need for shielding the hydrophobic surfaces by chaperones. Additionally, preformation of a highly ordered homo-oligomeric supercomplex ensures a high avidity and an immediate high local concentration of the active small oligomers (or monomers) upon membrane binding, which is likely necessary for membrane attachment and rapid formation of protein networks on membrane surfaces involved in membrane repair and/or protection. The orientation of the monomers in the ring could support e.g. membrane binding if the interacting amino acid residues are positioned in a favorable orientation to the membrane.

### The IM30 Ring Is Crucial for Membrane Fusion

Besides the involvement of the IM30 ring structure in membrane protection, the ring seems to be mandatory for the membrane fusion activity of IM30. A membrane fusion protein requires strict control of its activity, as any uncontrolled fusion event is potentially detrimental for the cell because it could e.g. result in a loss of electrical and/or chemical gradients. This is especially relevant for the TM, as any leak by misdirected fusion reduces the proton gradient and the photosynthetic efficiency. Therefore, control of the IM30 activity *via* oligomer formation might be a potential solution. Another regulation mechanism is the dependence of the fusion process on Mg^2+^, which seems to activate IM30 rings (Heidrich et al., 2018).

The structural features of the IM30 ring structure seem to support the fusion mechanism directly. An oligomeric ring exposes two distinct sides of the monomers, which are orientated unidirectional, as suggested for IM30 (Saur et al., 2017). The opposing sides of the ring can consequently interact *e.g.* with two membranes, as it would be necessary for a membrane fusion activity. This would also be possible with a cylindrically shaped protein, but the hole inside the ring might be important for the formation of a fusion pore(-like structure). A recent model for the IM30-mediated membrane fusion suggests that fusion is initiated by the ring (Heidrich et al., 2017). As the protein binds negatively charged lipids (Hennig et al., 2015), IM30 might recruit such lipids when binding as a ring to a membrane surface. In the center of the ring, the concentration of the non-bilayer forming lipid MGDG becomes locally high, which might result in disruption of an ordered bilayer structure and initial fusion of two interacting membranes. Dissociation of the ring could then lead to lipid mixing, allowing the formation of a stable, now fused membrane (Heidrich et al., 2017). Moreover, a large protein complex would clearly facilitate the formation of a fusion pore at the TM. As the membrane is completely crowded with integral and peripheral membrane proteins (Kirchhoff et al., 2008a; Casella et al., 2017; MacGregor-Chatwin et al., 2019; Wietrzynski et al., 2019), it is hard to imagine that binding of a small protein could provide enough space needed for membrane-membrane contacts and subsequent membrane fusion. Instead, binding of a large ring complex that finally dissociates could generate a fusion platform on the membrane. We, therefore, suggest that the IM30 ring structure is mandatory for the membrane fusion function.

Taken together, the formation of IM30 rings might prevent uncontrolled membrane binding and simultaneously prealigns IM30 monomers for efficient membrane binding. Furthermore, IM30 rings are directly involved in membrane fusion, where Mg^2+^ binding is an additional activation step that renders the rings fusion competent.

## Where and When to Find Im30 Rings?

As discussed above, IM30 rings are indispensable for controlled membrane remodeling but are probably also generally useful to ensure proper activity of IM30, *i.e.* increase the local concentration of active small oligomers/monomers or shield them from unwanted interactions when not bound to the membrane. An intriguing question that arises from these assumptions is: When such rings are so important, why do we not see them *in vivo*? While IM30 clusters have been observed on TMs in cyanobacteria and chloroplasts (Zhang et al., 2012; Bryan et al., 2014; Zhang et al., 2016; Gutu et al., 2018), the supramolecular organization of IM30 within these structures is still enigmatic, and rings “sitting” on TMs have not been observed yet. The problem probably is not that IM30 rings are hard to find because of their size and shape. Other proteins of similar or even smaller size have been identified in cryo-TEM tomograms of *Synechocystis* recently (Rast et al., 2019). So the question probably is more: Where and when to find IM30 rings in living cells?

IM30 appears to be a protein with (at least) a dual function, *i.e.* a membrane remodeling and a membrane stabilizing/protecting function. These functions have to be separated spatiotemporally, as they would otherwise cancel out each other. Assuming that both functions have different requirements on the protein structure (as discussed above), IM30 rings are very short-living and can probably only be found on a membrane under certain specific conditions. However, both processes, membrane chaperoning and remodeling, potentially involve binding of IM30 rings to the membrane and ring dissociation, resulting in small membrane-bound oligomers or even monomers (**Figure 2**). Thus, chances to find IM30 rings on membrane surfaces are probably low. Only at the initial phase of a fusion event, *i.e.* when two adjacent membranes meet each other, rings may be found to connect these (**Figure 2**). This may only be observed at TM convergence zones under conditions where membrane remodeling is needed, e.g. when cells are shifted from the dark to light. Under HL/stress conditions, IM30 monomers and/or small oligomers will be found on the TM (Thurotte et al., 2017; Gutu et al., 2018; Junglas and Schneider, 2018) (**Figure 2**), so chances to find rings are also low. Yet, as discussed above, the oligomeric state of IM30 depends on the activity of molecular chaperones, which likely control assembly and disassembly of IM30 oligomers (**Figure 2**). Indeed, IM30 supercomplexes have been detected in cellular extracts of *Chlamydomonas rheinhardtii* chloroplasts when ATP was depleted (Liu et al., 2007). Thus, IM30 rings might only exist in detectable amounts under conditions of low ATP, e.g. as periodically observed at the (diurnal) dark to light transition in cyanobacteria and chloroplasts (Konno et al., 2012; Saha et al., 2016; Voon et al., 2018). During dark to light transition, where IM30 is necessary for unimpaired growth (Gutu et al., 2018), IM30 rings might be needed for TM remodeling. However, the oligomeric state of IM30 is probably also controlled by altered expression of chaperones, as observed e.g. for DnaK2 under HL, heat, hyperosmotic and salt stress in *Synechococcus elongatus* PCC 7942 and *Synechocystis* sp. PCC 6803 (Sato et al., 2007; Rupprecht et al., 2008). Under normal growth conditions (no increased chaperone expression), IM30 rings could display an inactive cytosolic storage form. These possibly represent the diffuse particles observed by Gutu et al. (2018). Thus, the cellular ATP levels will likely have only minor effects on the oligomeric state of IM30 when chaperones are not induced.

In summary, we believe that IM30 rings can barely be found on membranes in living cells as they likely represent a short-lived IM30 structure. Assembly and disassembly of the supercomplexes likely are highly controlled and meetcellular demands.

## Author Contributions

All authors wrote the manuscript.

## Funding

This work was funded by the Max-Planck graduate center at the Max Planck institutes and the University of Mainz.

## Conflict of Interest

The authors declare that the research was conducted in the absence of any commercial or financial relationships that could be construed as a potential conflict of interest.
